# C-reactive protein and risk of breast cancer: A systematic review and meta-analysis

**DOI:** 10.1038/srep10508

**Published:** 2015-05-22

**Authors:** Lanwei Guo, Shuzheng Liu, Shaokai Zhang, Qiong Chen, Meng Zhang, Peiliang Quan, Jianbang Lu, Xibin Sun

**Affiliations:** 1 Department of Cancer Epidemiology, Affiliated Cancer Hospital of Zhengzhou University, Henan Cancer Hospital, Henan Office for Cancer Control and Research, Zhengzhou, China

## Abstract

Associations between elevated C-reactive protein (CRP) and breast cancer risk have been reported for many years, but the results remain controversial. To address this issue, a meta-analysis was therefore conducted. Eligible studies were identified by searching the PubMed and EMBASE up to December 2014. Study-specific risk estimates were combined using a random-effects model. Altogether fifteen cohort and case-control studies were included in this meta-analysis, involving a total of 5,286 breast cancer cases. The combined *OR* per natural log unit change in CRP for breast cancer was 1.16 (95% *CI*: 1.06-1.27). There was moderate heterogeneity among studies (*I*^2^ = 45.9%). The association was stronger in Asian population (*OR* = 1.57, 95% *CI*: 1.25-1.96) compared to European (*OR* = 1.12, 95% *CI*: 1.02-1.23) and American (*OR* = 1.08, 95% *CI*: 1.01-1.16). Prediagnostic high-sensitivity CRP concentrations (*OR* = 1.22, 95% *CI*: 1.10-1.35) was superior to common CRP (*OR* = 1.08, 95% *CI*: 1.01-1.15) in predicting breast cancer risk. The meta-analysis indicated that elevated CRP levels was associated with increased risk of breast cancer. Further research effort should be performed to identify whether CRP, as a marker of inflammation, plays a direct role in breast carcinogenesis.

Breast cancer is the second most common cancer worldwide and, by far, the most frequent cancer among women with an estimated 1.67 million new cancer cases diagnosed in 2012 (25% of all cancers)^1^. Although early diagnosis has contributed to the success of therapy, breast cancer remains a major problem of women’s health and its incidence is increasing in developing countries^2^. Since 1863 when Virchow hypothesized that cancer originated at the sites of chronic inflammation, a large number of experimental and epidemiological data has reinforced that chronic inflammation plays an important role in various aspects of cancer, including cancer initiation, promotion, progression, metastasis and clinical features^3^,^4^, all of which are hypothesized to be closely related to breast cancer development.

C-reactive protein (CRP) is a sensitive and widely used systemic marker of inflammation, which is mainly produced in the liver along with other acute-phase proteins in response to cytokines, such as Interleukin-6 (IL-6), IL-1, and Tumor Necrosis Factor-α (TNF-α)^5^. Compared with other inflammatory cytokines, CRP has several advantages in epidemiologic studies as a chronic inflammation marker, such as the availability of reliable assays and temporal stability^6^,^7^. Notably, elevated levels of CRP have been associated with several chronic diseases like overall cancer risk and risks of lung, colorectum, endometrium, and ovarian cancers^8^,^9^. However, data evaluating the association between CRP and breast cancer risk is rare and inconsistent.

During the last decade, several epidemiologic studies have appraised the associations between CRP and breast cancer risk. Thereinto, a meta-analysis published in 2009 found that a natural log (ln) unit increase in CRP was not statistically significant associated with breast cancer risk (relative risk [*RR*] = 1.10, 95% confidence interval [*CI*]: 0.97–1.26). However, significant heterogeneity was also found (*I*^2^ = 51.0%), and the estimation was based on only 1,240 breast cancer cases. Several epidemiologic studies with large sample size or long-term follow-up was performed thereafter. Therefore, a meta-analysis of cohort studies and case-control studies was conducted to further clarify the association between the elevated levels of CRP and breast cancer risk.

## Results

### Literature Search

**Figure 1:**
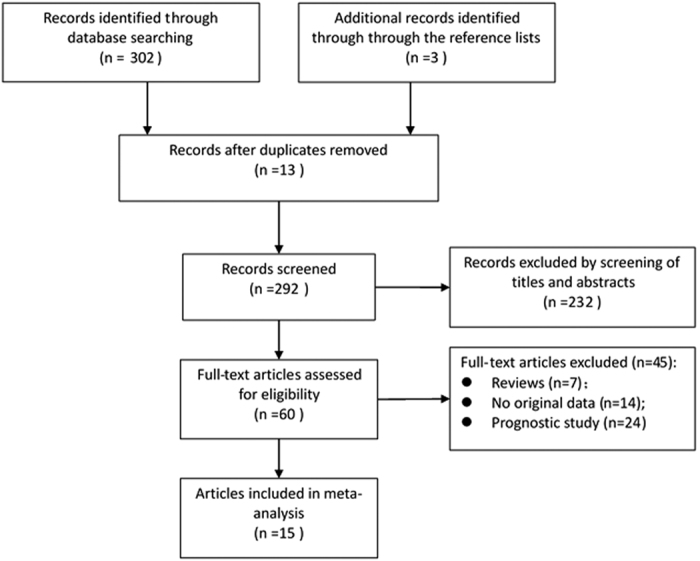
Flow diagram of systematic literature search.

As shown in Fig. 1, the search strategy generated 305 citations, of which 60 were considered potentially valuable after reading titles and abstracts, then the full text was retrieved for detailed evaluation, 45 were subsequently excluded for various reasons, including 7 were reviews, 14 that did not provide *OR*s or *CI*s and 24 were prognostic study. Eventually, 15 studies were included^8^,^10^,^11^,^12^,^13^,^14^,^15^,^16^,^17^,^18^,^19^,^20^,^21^,^22^,^23^.

### Characteristics of the selected studies

Individual characteristics of the included 15 studies (8 cohort studies, 5 nested case-control studies and 2 case-control studies) were summarised in Table 1. They were published from 2005 to 2014 and summed to 5,286 breast cancer cases totally. Six studies^13^,^15^,^16^,^17^,^21^,^22^ were conducted in the United States, six^8^,^10^,^12^,^18^,^19^,^20^ in Europe, and three^11^,^14^,^23^ in Asia. Incident cancers of six studies^8^,^10^,^13^,^16^,^17^,^20^ were ascertained by linkage to cancer registries, five^11^,^12^,^15^,^18^,^23^ by pathology reports, three^14^,^19^,^22^ by medical records and one^21^ was not given. Seven studies^8^,^10^,^11^,^17^,^18^,^19^,^23^ used CRP assays with high sensitivity; five studies^11^,^13^,^14^,^15^,^19^ used an enzyme linked immunosorbent assay (ELISA) to measure CRP, five^8^,^10^,^16^,^20^,^23^ used nephelometric assay, one^18^ used rate near-infrared particle immunoassay, three^12^,^17^,^22^ used immunoturbidimetric assay, and one^21^ used the Behring NA Latex test. Most studies provided risk estimates that were adjusted for age (12 studies), BMI (10 studies) and smoking (8 studies); fewer were adjusted for hormone replacement therapy (HRT) use (6 studies) and alcohol consumption (6 studies).

### Results of the meta-analysis

#### CRP and breast cancer

**Figure 2:**
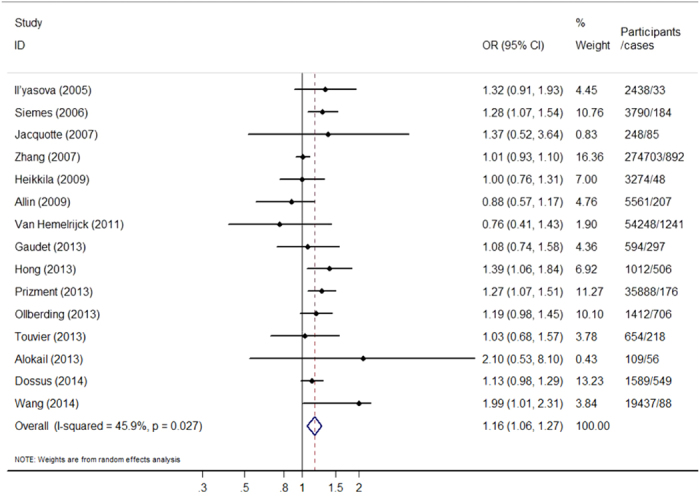
Forest plot for the association between per log-transformed CRP concentration and female breast cancer risk.

The multivariable-adjusted *OR*s for each study and all studies combined for one unit change in ln(CRP) were shown in Fig. 2. Among the 15 studies included, two showed an insignificant negative association between one unit change in ln(CRP) and breast cancer, and the other thirteen showed positive association, four of which showed statistical significance. The combined *OR* per natural log unit change in CRP for breast cancer was 1.16 (95% *CI*: 1.06-1.27). However, there was moderate heterogeneity observed across studies included (Q-test *P*_heterogeneity_ = 0.027, *I*^2^ = 45.9%).

#### Subgroup analyses

To explore the heterogeneity among studies of one unit change in ln(CRP) and breast cancer, we performed subgroup analyses (Table 2). The associations of ln(CRP) with breast cancer risk did not differ by study type, geographic region, CRP markers and CRP assay methodology, however, the association disappeared when stratified by BMI category. The association was stronger in retrospective case-control studies (*OR* = 1.42, 95% *CI*: 1.08-1.85) than in cohort studies and nested case-control studies (*OR* = 1.14, 95% *CI*: 1.04-1.25). The combined *OR* for breast cancer was 1.12 (95% *CI*: 1.02-1.23) for studies conducted in Europe, and 1.08 (95% *CI*: 1.01-1.16) in USA and 1.57 (95% *CI*: 1.25-1.96) in Asia. Elevated CRP levels significantly increased the risk of postmenopausal breast cancer (*OR* = 1.08, 95% *CI*: 1.00-1.16), but not significantly for premenopausal breast cancer (*OR* = 1.08, 95% *CI*: 0.91-1.28). Stratifying results by CRP markers showed that high-sensitivity CRP (*OR* = 1.22, 95% *CI*: 1.10-1.35) had a stronger association than common CRP (*OR* = 1.08, 95% *CI*: 1.01-1.15). And when stratified by CRP assay methodology, the combined *OR* was 1.25 (95% *CI*: 1.05-1.49) for CRP levels measured by ELISA assay, and 1.14 (95% *CI*: 1.03-1.27) by other assay. When cancer cases stratified by case diagnosis method, the association was significant for cases reported by cancer registry (*OR* = 1.13, 95% *CI*: 1.02-1.26) and pathology reports (*OR* = 1.23, 95% *CI*: 1.11-1.37), but not by medical records (*OR* = 1.04, 95% *CI*: 0.96-1.12).

### Influence analysis of individual studies

**Figure 3:**
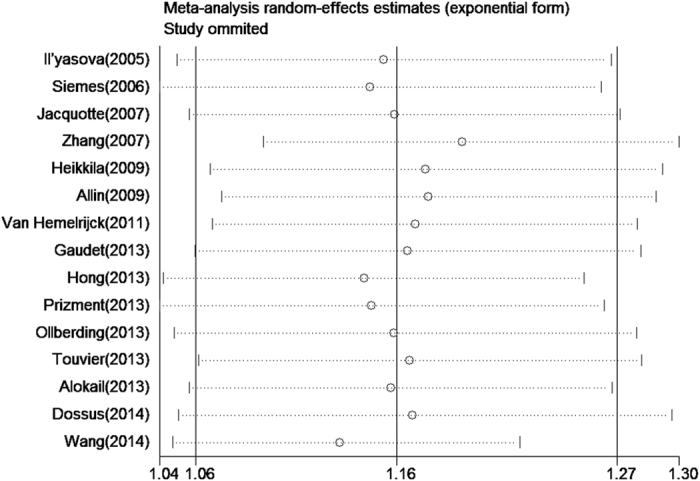
Influence analyses for omitting individual study on the summary odds ratio.

To address the potential bias due to the quality of the included studies, we performed the sensitivity analysis by calculating combined *OR* again when omitting one study at a time. Fig. 3 showed the results of sensitivity analysis. The combined *OR* per natural log unit change in CRP ranged from 1.13 (95% *CI*: 1.05-1.22) to 1.19 (95% *CI*: 1.09-1.30). The meta-analysis result of the combined *OR* per natural log unit change in CRP for breast cancer was not significantly affected by omission of any of the 15 individual studies, which meaned that each single study didn’t influence the stability of combined *OR* estimate.

### Publication bias

**Figure 4:**
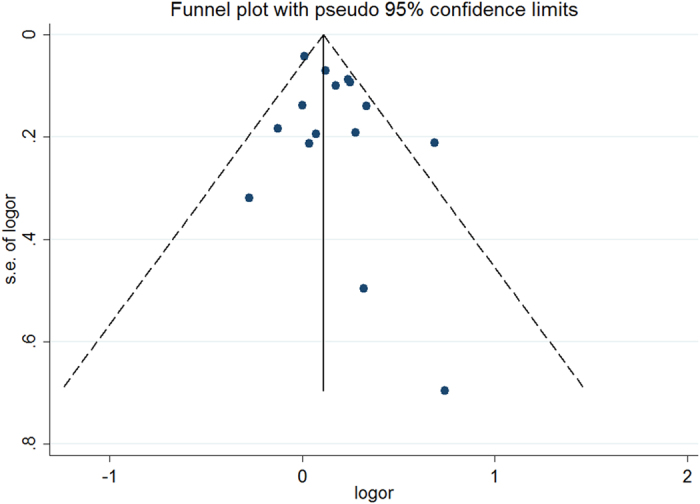
Funnel plot for analysis results of publication bias.

There was no evidence of publication bias as demonstrated by the non-significant *P* values for Begg’s (0.805) and Egger’s tests (0.172) and the near-symmetric funnel plot (Fig. 4).

## Discussion

This meta-analysis assessed the association between CRP levels and breast cancer risk. Overall, the result supported a significant positive association between the elevated levels of CRP and an increased risk of breast cancer. The overall estimate indicated an 16% increase in risk of breast cancer for a natural log unit increase in CRP levels. Sensitivity analysis further confirmed the robustness of results.

Our summary estimate of CRP and breast cancer risk in cohort studies was similar to that of another meta-analysis, which included 5 prospective studies with only 1,240 cases and reported a unit increase in ln(CRP) was associated with 10% increase in breast cancer risk. However, the result was not statistically significant and considerable heterogeneity was found (*I*^2^ = 51.0%). In contrast to that study, our meta-analysis enlarged breast cancer cases to 5,286 and the summary risk estimate showed smaller heterogeneity (*I*^2^ = 45.9%).

Results from subgroup analyses showed that geographic region, menstrual status, CRP markers and case diagnosis method might be possible sources of heterogeneity. Despite suffering the limitations of observational nature, several findings from subgroup-analysis deserved notable. A higher combined *OR* per natural log unit change in CRP was found in participants from Asia, which showed that regional differences might exist between the elevated levels of CRP and an increased risk of breast cancer. Results from subgroup analyses stratified by source of menstrual status showed that the elevated levels of CRP could increase the postmenopausal breast cancer, not the premenopausal breast cancer. As we all know, excess weight and obesity convincingly increase the risk of breast cancer in postmenopausal women^24^,^25^ and are established factors that contribute to chronic inflammation^26^. Despite the strong relationship between CRP and body weight^27^,^28^, the association between CRP levels and breast cancer risk was unlikely to be confounded by BMI, since four of six studies provided risk estimates that were adjusted for BMI. Besides, Hs-CRP, as an inflammatory biomarker, was superior to common CRP in predicting risk of breast cancer.

The present study has several strengths. First, it included a large sample size (5,286 breast cancer cases). Moreover, more comparable dose-response relationship were created for each study, and subgroup analyses stratified by 7 different variants were conducted, thus the effect of potential confounders was minimized. In addition, the combined *OR* per natural log unit change in CRP for breast cancer was not significantly affected by omission of any of the 15 individual studies, as well as no publication bias was observed in our analyses, indicating that our results were robust.

However, the present meta-analysis has several limitations. First, studies included in this meta-analysis were heterogeneous, which could be explained by differences in populations, CRP markers, and CRP detection method. To address this issue, the random-effects model meta-analysis was reported to combine data whenever significant heterogeneity was noted. We used appropriate well-motivated inclusion criteria to maximize homogeneity, and performed sensitivity and subgroup analyses to investigate potential sources of heterogeneity. Second, information was limited for the results stratified by menstrual status and BMI categories as not all studies involved here provided relevant information. Finally, a meta-analysis is not able to solve problems with confounding factors that may be inherent in the included studies. Although all the included studies presented here were carefully adjusted for potential confounders, including age, BMI, physical activity, smoking, alcohol consumption, HRT use, nonsteroidal anti-inflammatory drug (NSAID) use, it is possible that the associations of circulating CRP with breast cancer risk have been inflated by residual confounding or reverse causality. Insufficient control for confounding factors can skew the results in either direction, to exaggeration or underestimation of risk estimates. Besides, although it has been demonstrated that CRP levels are relatively stable over short periods of time and have little or no diurnal variation^29^, CRP levels are easily influenced by a variety of physiological and pathological stimulus, such as acute or chronic infection and use of anti-infectious agents. An alternative way to eliminate reverse causality and to minimize residual confounding would be to investigate the associations of breast cancer with genetic variants known to be associated with circulating CRP. As genetic variants are randomly allocated at conception, such investigations would provide unconfounded and unbiased estimates of any associations of inflammatory markers and any cancer outcomes^30^,^31^.

In conclusion, the findings of this meta-analysis indicated that elevated CRP levels was associated with increased risk of breast cancer, especially among the Asian population. Although causality evidence was insufficient, these results seemed to support a role of chronic inflammation in breast carcinogenesis. Further studies, especially with high-quality and more breast cancer cases involved cohort studies, are needed to identify whether CRP, as a marker of inflammation, does play a direct role in breast carcinogenesis.

## Methods

### Literature search strategy

A systematic search up to December of 2014 was conducted in MEDLINE (via PubMed) and Excerpta Medica database (EMBASE) to identify relevant articles. Search terms included “C-reactive protein” or “C reactive protein” or “CRP” combined with “breast cancer”. Additional relevant references cited in retrieved articles were also evaluated.

### Inclusion and exclusion criteria

All papers were reviewed by two authors independently. Uncertainties and discrepancies were resolved by consensus after discussing with a senior researcher. All studies included in the final meta-analysis satisfied the following criteria: (a) cohort or case-control study design; (b) report results on blood CRP levels; (c) breast cancer incidence as the outcome of interest; (d) report *RR* (or odds ratio [*OR*] estimates in case-control studies) or hazard ratios (*HR*) estimates with their corresponding 95% *CI* (or sufficient data to calculate of these effect measure). If the study was reported in duplication, the one published earlier or provided more detailed information was included. Review articles and editorials were included if they contained original data. Abstracts were excluded.

### Data extraction

Two of the authors performed the data extraction from each article and discrepancies were resolved by consensus. For studies meeting inclusion criteria, a standardized data extraction form was used to extract the following data: the first author’s name, year of publication, country of origin, study design, cohort study name, participants enrolled criteria, period of enrollment, the length of follow-up for cohort study, the number of participants (or person-years) and cancer cases, participants characteristics (gender composition, mean age, mean body mass index [BMI], menstrual status when blood was collected), CRP measurement methods, and *RR* or *OR* estimates with corresponding 95% *CI*s for CRP as a continuous variable or at least 3 categories of CRP levels. For each study, we extracted the risk estimates that were adjusted for the greatest number of potential confounders.

### Statistical analysis

The *RR* or *OR* per natural log unit change in CRP with 95% *CI* was used to compute the combined *OR* of elevated CRP levels and the risk of breast cancer. A fix-effect or random-effect model was used to combine the data, based on the Mantel–Haenszel method^32^ and the DerSimonian and Laird method^33^, respectively. These two models provide similar results when between-studies heterogeneity is absent; otherwise, random-effect model is more appropriate. For studies reporting no risk estimate for one unit change in ln(CRP), we used the method proposed by Orsini^34^ and Greenland^35^ to estimate the ln(*RR*) or (*OR*) for one unit increase in ln(CRP).

Cochrane *Q* test *(P* < 0.10 indicated a high level of statistical heterogeneity) and *I*^2^ ( values of 25%, 50% and 75% corresponding to low, moderate and high degrees of heterogeneity, respectively) was used to assess the heterogeneity between eligible studies, which test total variation across studies that was attributable to heterogeneity rather than to chance^36^. Subgroup analyses for one unit increase in ln(CRP) and the risk of breast cancer were subsequently carried out by study type, geographical region, menstrual status, BMI categories, CRP markers, CRP assay methodology and case diagnosis method. Sensitivity analysis was also conducted to assess the influence of each individual study on the strength and stability of the meta-analytic results. To show each study’s independent impact on the combined effect, only one study in the meta-analysis was excluded each time. Funnel plots and statistical tests (Begg adjusted rank correlation test and Egger regression asymmetry test) for funnel plot asymmetry were performed to test any existing publication bias.

All statistical analyses were performed using STATA version 12 for Windows (StataCorp LP, College Station, TX, USA). A two-tailed *P* < 0.05 was considered statistically significant.

## Additional Information

**How to cite this article**: Guo, L. *et al*. C-reactive protein and risk of breast cancer: A systematic review and meta-analysis. *Sci. Rep.* **5**, 10508; doi: 10.1038/srep10508 (2015).

## Figures and Tables

**Table 1:** Characteristics of the included studies.

**First author**	**Year**	**Study Year of recruitment**	**Country**	**Study design**	**Age, y**	**No. of Subjects/cases**	**Outcome assessment**	**Markers**	**CRP measurement methods**
Il’yasova	2005	HABCS 1997-1998	USA	Cohort	73(70-79)	2438/33	Pathology reports	CRP	ELISA
Siemes	2006	Rotterdam 1989-1993	Netherlands	Cohort	69.6(9.2)	3790/184	Pathology reports	Hs-CRP	Rate Near-infrared Particle Immunoassay
Jacquotte	2007	NYUWHS	USA	Nested C-C	Not Given	248/85	Not Given	CRP	The Behring NA Latex Test
Zhang	2007	WHS 1992	USA	Cohort	54.5	274703/892	Medical records	CRP	Latex-enhanced Immunoturbidimetry
Heikkila	2009	BWHHS 1999-2001	British	Cohort	69.2	3274/48	Cancer registry	Hs-CRP	Ultrasensitive Nephelometry
Allin	2009	CCHS 1946-1978	Danish	Cohort	30-71	5561/207	Cancer registry	Hs-CRP	Turbidimetry or Nephelometry
Van Hemelrijck	2011	AMORIS 1985-1986	Sweden	Cohort	44.8(16.68)	54248/1241	Cancer registry	CRP	Turbidimetry
Gaudet	2013	CPS-II 1998-2001	USA	Nested C-C	50-74	594/297	Cancer registry	CRP	ELISA
Hong	2013	2008-2011	China	C-C	53.7(12.1)	1012/506	Medical records	CRP	ELISA
Prizment	2013	ARIC 1987-1989	USA	Cohort	45-64	35888/176	Cancer registry	Hs-CRP	Immunoturbidimetry
Ollberding	2013	Multiethnic 2001-2006	USA	Nested C-C	67.8(7.4)	1412/706	Cancer registry	CRP	Turbidimetry
Touvier	2013	SU.VI.MAX 1994-1995	France	Nested C-C	49.2(6.1)	654/218	Medical records	Hs-CRP	ELISA
Alokail	2013	Not Given	KSA	C-C	46.4(11.3)	109/56	Pathology reports	Hs-CRP	ELISA
Dossus	2014	E3N 1995-1999	France	Nested C-C	57.6(6.1)	1589/549	Pathology reports	CRP	Particle-enhanced Immunoturbidimetry
Wang	2014	Kailuan 2006-2011	China	C-C	49.2(11.3)	19437/88	Pathology reports	Hs-CRP	Nephelometry

Abbreviations: HABCS, the Health Aging and Body Composition Study; NYUWHS, the New York University Women’s Health Study; WHS, the Women’s Health Study; BWHHS, the British Women’s Heart and Health Study; CCHS, the Copenhagen City Heart Study; AMORIS, the Apolipoprotein MOrtality RISk study; CPS-II, the American Cancer Society’s Cancer Prevention Study-II Nutrition Cohort; ARIC, the Atherosclerosis Risk in Communities Cohort Study; SU.VI.MAX, the Supplémentation en Vitamines et Minéraux Antioxydants Study; E3N, the E3N Cohort Study; CRP, C-reactive protein; Hs-CRP, High-sensitivity C-reactive protein; C-C, case-control; ELISA, enzyme linked immunosorbent assay.

**Table 2:** Results of subgroup analyses.

**Group**	**No. of study**	***OR*** **(95% *CI*)**	**Heterogeneity test**	
			***P*** **for** ***Q*** **test**	***I***^**2**^**, %**†
All	15	1.16 (1.06-1.27)	0.027	45.9
Study type				
Prospective*	13	1.14 (1.04-1.25)	0.033	46.4
Retrospective**	2	1.42 (1.08-1.85)	0.564	0.0
Geographic region				
Europe	6	1.12 (1.02-1.23)	0.298	17.9
USA	6	1.08 (1.01-1.16)	0.152	38.1
Asia	3	1.57 (1.25-1.96)	0.340	7.2
Menstrual status				
Premenopausal	2	1.08 (0.91-1.28)	0.551	0.0
Postmenopausal	6	1.08 (1.00-1.16)	0.208	30.3
BMI (kg/m^2^)				
< 25	3	1.09 (0.96-1.25)	0.408	0.0
≥ 25	4	1.41 (0.96-2.07)	0.003	78.1
Markers				
Hs-CRP	7	1.22 (1.10-1.35)	0.056	51.1
CRP	8	1.08 (1.01-1.15)	0.211	27.2
CRP assay methodology				
ELISA	5	1.25 (1.05-1.49)	0.623	0.0
Other assay	10	1.14 (1.03-1.27)	0.011	58.0
Case diagnosis method				
Cancer registry	6	1.13 (1.02-1.26)	0.280	20.3
Pathology reports	5	1.23 (1.11-1.37)	0.109	47.1
Medical records	3	1.04 (0.96-1.12)	0.091	58.4

Abbreviation: *OR*, odds ratio; *CI*, confidence intervals; BMI, body mass index; Hs-CRP, High-sensitivity C-reactive protein; ELISA, enzyme-linked immunosorbent assay.

^*^Refers to cohort study and nested case-control study;

^**^Refers to case-control study;

^†^*I*^2^ is interpreted as the proportion of total variation across studies that are due to heterogeneity rather than chance.
